# Treating Mucocele in Pediatric Patients Using a Diode Laser: Three Case Reports

**DOI:** 10.3390/dj6020013

**Published:** 2018-05-09

**Authors:** Sara M. Bagher, Ayman M. Sulimany, Martin Kaplan, Cheen Y. Loo

**Affiliations:** 1 Pediatric Dentistry Department, King Abdulaziz University, Jeddah 21589, Saudi Arabia; 2 Department of Pediatric Dentistry and Orthodontics, College of Dentistry at King Saud University, Riyadh 115451, Saudi Arabia; a.sulimany@windowslive.com; 3 Department of Pediatric Dentistry, Tufts University School of Dental Medicine, 1 Kneeland Street, Boston, MA 02111, USA; martin.kaplan4@verizon.net (M.K.); c.loo@tufts.edu (C.Y.L.)

**Keywords:** diode laser, mucocele, pediatric patient

## Abstract

A mucocele is the most common minor salivary gland disease and among the most common biopsied oral lesions in pediatric patients. Clinically, a mucocele appears as a round well-circumscribed painless swelling ranging from deep blue to mucosa alike in color. Mucoceles rarely resolve on their own and surgical removal under local anesthesia is required in most cases. Different treatment options are described in the literature, including cryosurgery, intra-lesion injection of corticosteroid, micro-marsupialization and conventional surgical removal using a scalpel, and laser ablation. Therefore, the goal of this paper was to report three cases of mucocele removal in pediatric patients using a diode laser with a one-month follow-up. Mucoceles were removed by a pediatric dentist using a diode laser with a wavelength of 930 nm in continuous mode and a power setting of 1.8 Watts. In all cases, no bleeding occurred during or after the procedure and there was no need for suturing. On clinical examination during the one-month follow-up, in all three cases there was minimal or no scarring, minimal post-operative discomfort or pain, and no recurrence. Diode lasers provide an effective, rapid, simple, bloodless and well accepted procedure for treating mucocele in pediatric patients. Minimal post-operative discomfort and scarring was reported by all the three patients.

## 1. Introduction

A mucocele is considered the most common minor salivary gland disease [1] and among the most common biopsied oral lesions in pediatric patients [2,3]. It affects both genders [1], with peak incidence among children and young adults [1,4,5]. It is caused by accumulation of mucus that spills from the salivary glands and their ducts into the oral cavity’s subepithelial tissue [1]. Clinically, it appears as a round, well-circumscribed, painless swelling that ranges from deep blue to mucosa-like in color [1,5]. Mucoceles are usually asymptomatic, though in some patients they can cause discomfort and interfere with speech, chewing and swallowing [1,6]. The lower lip is the most common site for a mucocele followed by the cheeck mucosa and the floor of the mouth [4,7,8].

Based on etiology, mucoceles are classified as retention and extravasation mucoceles [1,4,6]. Extravasation mucoceles are considered pseudo-cysts with no epithelial lining and caused by trauma to the excretory duct of minor salivary gland, followed by rapture of the duct causing extravasation and accumulation of saliva in the surrounding connective tissue [1,4,5]. Extravasation mucoceles are usually found in the lower lip of younger patients [1,5] and account for over 80% of all mucoceles [1,6]. In contrast, retention mucoceles are true cysts with cubic or squamous cell epithelial linings [1]. They are less common and caused by ductal obstruction that interferes with the normal salivary flow causing mucosal swelling and ductal dilatation [1,6]. 

Mucoceles rarely resolve on their own and surgical removal is required in most cases [1,5,9], which can be challenging, especially in children and patients with behavioral problems. The literature describes different treatment options, including cryosurgery [10,11], intra-lesion injection of corticosteroid [9], micro-marsupialization [12], conventional surgical removal [1,5,13], and laser ablation [14,15,16]. 

The conventional surgical removal of mucoceles using a scalpel is considered the most common option and requires complete resection of the mucocele and the neighboring glands before closure to decrease the risk of relapse [6]. Only limited studies have been published about laser mucocele removal in adults [15,16,17] and pediatric patients [7,8,16,18], and they reported that using a laser is a rapid, simple, bloodless procedure with minimal scarring and postoperative discomfort and recurrence compared to conventional surgical excision. Therefore, the goal of this study was to report three clinical cases of surgical removal and one-month follow-up of mucocele treatment in pediatric patients using a diode laser. 

## 2. Case Reports

**Figure 1:**
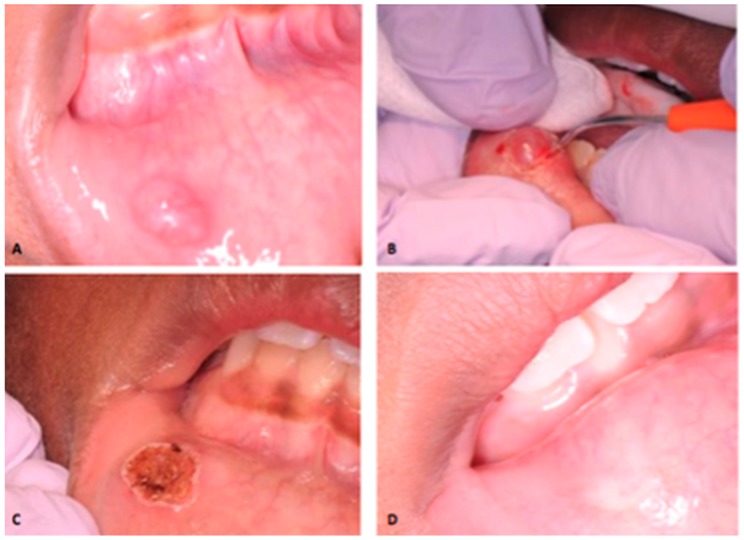
Laser removal of mucocele of the lower lip in an eight years old female. (**A**) Initial clinical presentation. (**B**) Removal of mucocele by use of high-intensity diode laser. (**C**) Immediate postoperative view. (**D**) Clinical appearance after one month.

**Case 1:** An eight-year-old, healthy, African-American female patient presented with her mother at the department of Pediatric Dentistry at Tufts University School of Dental Medicine (TUSDM) complaining of asymptomatic swelling in the labial mucosa of her lower lips. No significant medical history or known allergies were reported. Examination revealed a 0.70 cm silver blue, translucent swelling opposite the right mandibular canine. The mother reported that the swelling started four months before and changed episodically in size and color. They denied any previous trauma or habit of lip biting (Figure 1). 

**Figure 2:**
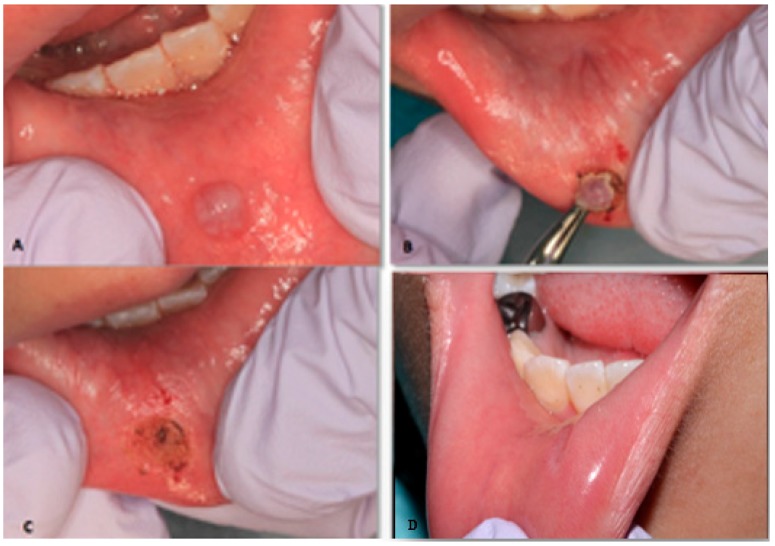
Laser removal of mucocele of the lower lip in an eight years old female. (**A**) Initial clinical presentation of the mucocele. (**B**) Mucocele removal by using high-intensity diode laser. (**C**) Immediate postoperative view. (**D**) Clinical appearance of the removed mucocele after one month.

**Case 2:** An eight-year-old, healthy, Asian female patient presented with her parents at the department of Pediatric Dentistry at TUSDM for initial dental examination. The patient complained of swelling in her lower lip. No significant medical history or known allergies were reported. Examination revealed a 2-cm translucent swelling in the labial mucosa on the lower lip opposite the left mandibular lateral incisor. Her history revealed that the swelling appeared a long time earlier and did not change in size and color. The parents denied any previous trauma or habit of lip biting (Figure 2). 

**Figure 3:**
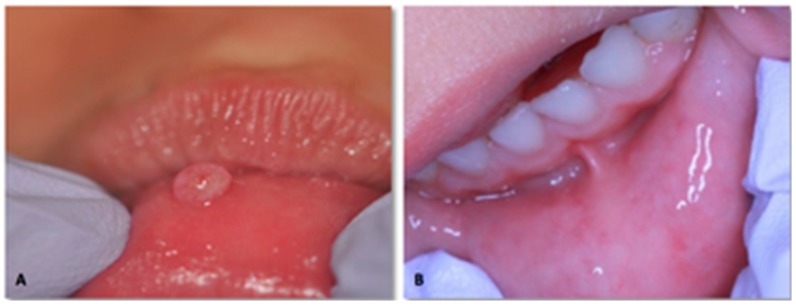
Laser removal of mucocele of the lower lip in a four years old male. (**A**) Initial clinical presentation (**B**) Clinical appearance after one month.

**Case 3:** A four-year-old, healthy Caucasian male patient presented with his parents at the department of Pediatric Dentistry at TUSDM for an emergency, complaining of asymptomatic swelling in the labial mucosa on the lower lip. No significant medical history or known allergies were reported. Examination revealed a 0.60 mm pale pink swelling opposite the right mandibular lateral incisor. It had begun several months before and the parents noticed that it increased in size. The parents confirmed that the patient was in the habit of biting his lip (Figure 3). All patients reported mild discomfort while eating and speaking.

## 3. Management 

The initial diagnosis of extravasation mucocele was established based on the lesion history and clinical findings. Treatment options were discussed with all patients and parents before an appointment was scheduled, and the risks and advantages of conventional and laser treatment also were described. 

The lesions were removed by the same pediatric dentist under local anesthesia using a diode laser with a wavelength of 980 nm in continuous mode at a power setting of 1.8 Watts (W) (DioDent Micro 980, Hoya Conbio, Fremont, CA, USA). First, a topical Benzocaine gel 20% anesthetic gel (Centrix, River Road Shelton, CT, USA) was applied for 2 min, followed by infiltration around the periphery of the lesion with Lidocaine 2% with 1:100,000 epinephrine (DENTSPLY, College Ave, York, PA, USA). Following the manufacturer’s instructions, the fiber tip was moved across a piece of articulating paper with the unit set to one W to accomplish the initiation of the fiber to become a useful hot-tip-thermal 600 °C contact device (without initiation there would be no useful clinical effect). The patient, operating pediatric dentist, and the assistant all wore safety laser-specific eye wear during the procedure. The staff also wore laser plume safety masks. After the initiation, mucocele excision was performed by separating the lesion and its associated minor salivary gland from the adjacent mucosa A tissue holding forceps was used to retract the mucocele tissue to one side carefully with laser beam parallel to the long axis of the tissue to avoid damaging the adjacent tissues.

Intermittently, water cooling with water-moistened gauze was used to control tissue temperature and to remove any tissue debris and char adhering to the hot fiber tip. High speed air suction of the tissue plume and air cooling over the treatment site was also employed. There was no bleeding during or after the procedure in any of the cases and the wounds were left open without suture. No antibiotics or anti-inflammatory analgesics were prescribed. The excised lesions were placed in water for two minutes and then were stored in 10% formalin and sent to the Department of Oral Pathology at TUSDM. The results confirmed the initial diagnosis.

All patients were advised not to consume hot, spicy food for a day and to rinse with normal saline mouthwash three times daily for the following five days. The patient with a history of lip biting was advised not to bite his lower lip. Parents were instructed to come for follow-up visits two weeks and one month after the procedure. The parents were contacted by phone the same day and two days after the procedure to evaluate the post-operative discomfort and pain felt by the patient. At the one-month follow-up, examination revealed minimal or no scarring and no recurrence in all cases; all patients reported minimal post-operative discomfort and pain and none of them required post-operative pain medication.

## 4. Histological Report 

Microscopic examination revealed a soft tissue lesion covered by a stratified mucous epithelium. The lamina propria consisted of fibrous connective tissue that was replaced focally by a superficial pool of mucin and surrounded by a granulation tissue wall. Bolus of minor salivary gland with chronic inflammation and dilated duct was also reported in the specimen from the third case. The histologic diagnosis confirmed the initial diagnosis of extravasation mucocele. 

## 5. Discussion 

This paper reports the removal of three cases of mucoceles in pediatric patients using diode laser with a one-month follow-up Laser ablation [14,15,16] is one of the treatment modalities for the removal of mucocele. Various types of lasers have been used to treat and remove mucoceles such as carbon dioxide (CO_2_) [7,8,16], erbium [19] and diode laser [18,20,21].

The laser diode was first introduced in dentistry in the mid 1990s. It is manufactured from semiconductor crystals with a short-wave length (800–980 nm, and most recently, 1064 nm) and works by transmitting photo-thermal energy to cells it contacts that causes an increase in temperature, protein denaturation, vaporization, and carbonization [12,22]. A diode laser has high affinity to hemoglobin and melanin causing an elevation in the temperature and promoting coagulation and hemostasis [22,23].

Small, inexpensive, portable machines are considered among the advantages of a diode laser by comparison to other lasers [23]. A diode laser delivers the energy fiber photothermally in contact with the soft tissue. The fiber optic tip needs to be initiated to focus the laser energy at the contact point into thermal energy and accelerate tissue incisions [23]. The thermal damage zone on the borders of the excisional biopsies is significantly larger with diode laser compared to CO_2_ laser [24]. Therefore, to control the thermal effect, the tissue temperature is regulated using air and water to cool the surgical site. Further, setting the wattage properly and using continuous or gated pulsing parameters are recommended when using a diode laser to control the thermal effects on the soft tissue [18,25]. 

Mucocele removal with laser provides rapid, bloodless procedure, with minimal if not bleeding, swelling, scaring and post-operative discomfort [7,8,15,16,17,18]. Only limited studies have been published regarding laser mucocele removal in pediatric patients [7,8,16,18] and no bleeding, no suture and time-saving were the main advantages as reported by these studies. Huang et al., in 2003 reported that the mean time required to perform the procedure was three to five minutes. This makes mucocele laser removal more suitable than a bladed surgical procedure especially for children and less cooperative patients.

At the one-month follow-up, clinical examination revealed no scar formation and all patients reported minimal post-operative discomfort and pain. These results coincide with the findings in previous case reports [18,20]. In 2010, Pedron et al. reported that the removal of mucocele using a diode laser in pediatric patients is simple, fast, rapid, bloodless, and tolerated well. One-month follow-ups revealed minimal postoperative problems, discomfort, and scarring [18]. In 2015, Pagila et al., reported a case of mucocele removal using a diode laser in a three-month-old infant. Two weeks later, the follow-up showed that the wound had healed perfectly [20]. Recently, Ramkumar et al., reported a case of mucocele excision using a diode laser in a 16-year-old patient. The author reported that the reduced duration of the procedure, good visualization, and hemostasis were the main advantages of using a diode laser [26]. Despite the limited number of cases presented and the short-term follow-up of this case report, the use of a diode laser appears to present a good alternative treatment to remove mucocele in pediatric patients. However, more clinical studies with larger sample sizes and longer follow-up periods are warranted in attempts to improve the management of oral mucoceles using a diode laser.

## 6. Conclusions

Diode lasers can be used on pediatric patients to remove mucocles. It provides an effective, rapid, simple, bloodless and well-accepted procedure for treating mucocele in pediatric patients. In addition, minimal post-operative discomfort and scarring were reported by all the cases presented.
